# Psychiatric comorbidities and photophobia in patients with migraine

**DOI:** 10.1186/s10194-017-0718-1

**Published:** 2017-02-09

**Authors:** Stefan Seidel, Roland Beisteiner, Maike Manecke, Tuna Stefan Aslan, Christian Wöber

**Affiliations:** Department of Neurology, Medical University of Vienna, Währinger Gürtel 18-20, A-1090 Vienna, Austria

**Keywords:** Migraine, Photophobia, Depression, Anxiety, Stress

## Abstract

**Background:**

Based on recent findings and our own impressions we took a closer look at the relationship between (inter)ictal photophobia and psychometric variables in migraine patients with photophobia.

**Findings:**

For this study we included 29 (27 female) migraine patients and 31 (18 female) controls with a mean age of 31.6 ± 12.5 years and 24.0 ± 4.1 years, respectively. All participants filled out the Depression Anxiety Stress Scale (DASS).

Interictal photophobia in patients was significantly higher than photophobia in controls (*p* = .001). Patients showed statistically significantly higher levels of depressive symptoms (*p* < .001), anxiety symptoms (*p* < .001) and stress (*p* < .001) than controls. Among all participants, (interictal) photophobia correlated positively with age (rho = .318, *p* = .013) as well as with the levels of depressive symptoms (rho = .459, *p* < .001), anxiety symptoms (rho = .346, *p* = .008) and stress (rho = .368, *p* = .005), but not with gender. In the patients, ictal photophobia correlated positively with age (rho = .473, *p* = .01) and interictal photophobia (rho = .423, *p* = .022). Linear regression analysis revealed only a trend towards statistical significance for (interictal) photophobia as a predictor for the level of depressive symptoms (rho = .457, *p* = 0.056) in the whole sample.

**Conclusions:**

Considering higher levels of photophobia in depression and the comorbidity of migraine and depression, it might be possible that depression contributes to interictal photophobia in patients with migraine. The same may be true for anxiety and stress. Both are also related to migraine and their possible impact on photophobia in migraine may be explained by pupillary dysfunction.

## Introduction

Photophobia is a common symptom of migraine attacks experienced by up to 80% of the patients. The prevalence seems to increase with age [1–3]. Sensitivity to light is not limited to the headache phase, but frequently is also present in the premonitory phase as well as after headache has subsided [1]. Measuring quantitative thresholds for discomfort and pain with monocular and binocular light stimuli, Vanagaite et al. [4] found that migraine patients were more photophobic during attacks than outside attacks and that they were more sensitive to light than controls even between attacks. Main et al. [5] reported significantly lower interictal light discomfort thresholds in migraineurs compared to controls. Functional neuroimaging showed more activation of the extrastriate visual cortex in migraine patients with photophobia compared to those without photophobia during the premonitory phase of a migraine attack [6, 7].

The comorbidity of migraine with depression, anxiety and stress is well known and was investigated recently in a population-based study by Risal et al. which highlighted the negative consequences of these co-morbidities on the quality of life in migraine patients [8]. In this context it appears noteworthy that patients with psychiatric disorders often exhibit affective temperament dysregulation and suicidal behaviors [9]. These specific characteristics may significantly contribute to the psychocial impairment and altered quality of life of these subjects. A possible relation between depression and photophobia has been reported already in 1989 [10]. Recently Llop et al. [11] and Anagnostou et al. [12] investigated the association of migraine and photophobia. While the former authors [11] found that photophobia might predispose migraineurs for psychiatric comorbidities, the latter showed that photophobia may not exclusively be related to migraine [12].

During the patient recruitment for a case-control functional magnetic resonance imaging (fMRI) study on the effects of repeated flicker light exposure on photophobia in migraine patients (KLI 455, Austrian Science Fund, FWF) we noticed that the number of patients with significant psychiatric comorbidities exceeded our expectations.

Hence, we decided to take a closer look at the relationship between (inter)ictal photophobia and psychometric variables in the subjects screened for participation in our fMRI study.

## Findings

We included patients with migraine and migraine-free controls screened for possible participation in a prospective study on the effects of repeated flicker light exposure on the level of photophobia (KLI 455, Austrian Science Funds (FWF)).

The participants’ age had to be between 18 to 45 years. Inclusion criteria for patients comprised (1) migraine without aura according to the criteria of the beta-version of the third edition of the International Classification of Headache Disorders (ICHD-3 beta) [13], (2) 1–4 days with migraine per month in the 3 months preceding study inclusion, (3) an intensity of interictal photophobia of 2–6 on a numeric rating scale (NRS, range 0 to 10) and (4) an intensity of ictal photophobia of >4. Controls had to fulfil the following inclusion criteria: (1) no personal or family history of migraine and (2) photophobia <2 on the NRS. Exclusion criteria for both groups were: (1) any other recurrent current or previous headache disorder apart from infrequent tension-type headache, and (2) current or previous medication overuse. All participants completed the 21-item version of the Depression Anxiety Stress Scale (DASS) [14]. It consists of three self-report scales (7 items per scale) designed to measure the negative emotional states of depression, anxiety and stress. The depression scale assesses dysphoria, hopelessness, devaluation of life, self-deprecation, lack of interest/involvement, anhedonia, and inertia. The anxiety scale assesses autonomic arousal, skeletal muscle effects, situational anxiety, and subjective experience of anxious affect. The stress scale is sensitive to levels of chronic non-specific arousal. It assesses difficulty relaxing, nervous arousal, and being easily upset/agitated, irritable/over-reactive and impatient. Subjects are asked to use 4-point severity/frequency scales to rate the extent to which they have experienced each state over the past week. Scores for depression, anxiety and stress are calculated by summing the scores for the relevant items. Paricipants were excluded from the flicker light study, if the DASS depression subscore was ≥7 points and/or the anxiety subscore was ≥6 points.

So far, we have screened 29 (27 female) migraine patients and 31 (18 female) controls (*p* = .005) with a mean age of 31.6 ± 12.5 years and 24.0 ± 4.1 years (*p* = .74), respectively. We were astounded that 10 (40%) migraine patients had to be excluded from the flicker light study, because the DASS depression subscore was ≥7 and/or the anxiety subscore was ≥6, while none of the controls exceded these cut-off scores (*p* < 0.001).

Interictal photophobia was <2, 2–6, and >6 in 7, 19, and 3 patients; ictal photopobia was ≤4 in 1 and >4 28 patients. Ten controls gave photophobia ≥2. Interictal photophobia in patients was significantly higher than photophobia in controls (Table 1, *p* = .001). Patients and controls also differed statistically significantly regarding their levels of depressive symptoms (*p* < .001), anxiety symptoms (*p* < .001) and stress (*p* < .001) on the DASS (Table 1).

**Table 1:** Clinical data of migraine patients and controls

		Patients (*n* = 29)	Controls (*n* = 31)	*p*-value
Age	mean ± SD	31.6 ± 12.5	24.0 ± 4.1	.74
	range	19–68	20–29	
Gender (m:f)	n	2:27	12:18	.005^*^
(Interictal) photophobia (NRS)	mean ± SD	3.1 ± 2.1	1.5 ± 1.8	.001^+^
	range	0–8	0–7	
Ictal photophobia (NRS)	mean ± SD	7.3 ± 1.8	N/A	–
	range	3–7	N/A	
DASS depression score	mean ± SD	3.6 ± 4.0	0.7 ± 1.1	<.001^+^
	range	0–15	0–5	
DASS anxiety score	mean ± SD	4.0 ± 4.0	0.4 ± 0.8	<.001^+^
	range	0–14	0–3	
DASS stress sore	mean ± SD	6.0 ± 5.2	1.4 ± 1.9	<.001^+^
	range	0–19	0–7	

*DASS* Depression Anxiety Stress Scale, *N/A* not applicable, *NRS* numeric rating scale, *SD* standard deviation. ^+^Mann-Whitney-U-test; ^*^Chi-Quadrat-test

To further evaluate the impact of photophobia we performed multivariate correlation analyses including age, gender and DASS scores of depressive symptoms, anxiety symptoms and stress as well as linear regression analyses using each of the three psychiatric domains as dependent variables. Both calculations were done for the entire sample of participants (*n* = 60) and separately for patients with migraine (*n* = 29) and controls (*n* = 31).

Among all participants, (interictal) photophobia correlated positively with age (rho = .318, *p* = .013) as well as with the DASS subscores for depressive symptoms (rho = .459, *p* < .001), anxiety symptoms (rho = .346, *p* = .008) and stress (rho = .368, *p* = .005), but not with gender. In the patients, the only statistically significant correlations were those of ictal photophobia with age (rho = .473, *p* = .01) and interictal photophobia (rho = .423, *p* = .022). Ictal photophobia did not correlate with gender and DASS scores. Interictal photophobia did not show any statistically significant correlations in patients and the same was true for photophobia in controls.

Linear regression analysis revealed only a trend towards statistical significance for (interictal) photophobia as a predictor for the level of depressive symptoms (rho = .457, *p* = 0.056) in the whole sample. In separate analyses of patients and controls, none of the factors age, gender, (interictal) photophobia and ictal photophobia (for migraine patients only) significantly predicted the levels of depressive symptoms, anxiety symptoms and stress.

## Discussion

This analysis of 60 subjects screened for participation in a prospective study on the effects of repeated flicker light exposure on the level of photophobia revealed a trend towards a relation of photophobia to the severity of depressive symptoms, anxiety symptoms and stress. However, this was only true for the total group of migraine patients and controls and not for each of the two study groups separately.

Accordingly, we failed to replicate the findings of Llop et al. [11]. These authors applied the Beck Depression Inventory (BDI-II) and the Beck Anxiety Inventory (BAI) in migraine patients with and without interictal photophobia and in migraine-free controls and included 16 subjects in each of the three groups. BDI-II and BAI scores were statistically significantly higher in patients with interictal photophobia than in those without interictal photophobia and in migraine-free controls.

The conflicting findings in our study and that of Llop and co-workers [11] may be explained by the different questionnaires (DASS vs. BDI-II and BAI) and by differences in age (31.6 vs 51 years). The latter might be more relevant following the hypothesis of Llop et al. [11] that interictal photophobia may increase with age as part of a central sensitization process. Similarly Grassini and Nordin [15] hypothesized the central sensitization may explain depression, anxiety, stress, functional somatic syndromes, and somatization in patients with migraine.

Considering higher levels of photophobia in depression [16] and the comorbidity of migraine and depression [17], it might be possible that depression contributes to interictal photophobia in patients with migraine. The same may be true for anxiety and stress. Both are also related to migraine [18] and their possible impact on photophobia in migraine may be explained by pupillary dysfunction [18–20]. In anxiety disorders [18, 19] and posttraumatic stress disorder [20] exaggerated pupillary dilation in response to stressful and negative stimuli is a reliable autonomous marker, even beyond the clinically active phase of the condition [20]. In contrast, a most recent abstract [21] showed a sympathetically-mediated association between interictal photophobia and pupil size in patients with migraine. In this study, lower photophobia thresholds were related to smaller dark-adapted pupil size and impaired re-dilation following a brief pulse of light. Re-dilation was least pronounced in the most severely affected patients. These findings suggest a physiological coping mechanism to a lowered threshold for light sensitivity in patients with migraine. Recently, prolonged neuronal suppression after visual adaptation in migraine patients has been reported [22]. One may speculate that pupillary dilation related to anxiety and stress counteracts this coping mechanism and thus causes increased photophobia. Finally, tizanidine, a drug used in migraine prophylaxis, has been shown to reduce pain-induced pupillary dilation [23], but the effect of tizanidine on photophobia in migraine has not been assessed.

Our study is limited by the small sample size emphasizing the preliminary and exploratory nature of our main findings. It may also have been biased by the predominance of the female gender in the patient group compared to the controls. The inclusion of another control group consisting of migraine patients without interictal photophobia would have further strengthened our data.

In conclusion, the relation of photophobia, pupillary function, and psychiatric comorbidities in patients with migraine is not well understood and deserves further and larger studies.

## Abbreviations

DASS: Depression Anxiety Stress Scale
fMRI: functional magnetic resonance imaging
ICHD: International Classification of Headache Disorders
NRS: Numeric rating scale

## References

[1] Giffin NJ, Ruggiero L, Lipton RB, Silberstein SD, Tvedskov JF, Olesen J (2003). Premonitory symptoms in migraine: an electronic diary study. Neurology.

[2] Kelman L (2006). Migraine changes with age: IMPACT on migraine classification. Headache.

[3] Wöber-Bingöl C, Wöber C, Karwautz A, Auterith A, Serim M, Zebenholzer K (2004). Clinical features of migraine: a cross-sectional study in patients aged three to sixty-nine. Cephalalgia.

[4] Vanagaite J, Pareja JA, Storen O, White LR, Sand T, Stovner LJ (1997). Light-induced discomfort and pain in migraine. Cephalalgia.

[5] Main A, Dowson A, Gross M (1997). Photophobia and phonophobia in migraineurs between attacks. Headache.

[6] Maniyar FH, Sprenger T, Monteith T, Schankin CJ, Goadsby PJ (2015). The premonitory phase of migraine--what can we learn from it?. Headache.

[7] Maniyar FH, Sprenger T, Shankin CJ, Goadsby PJ (2014). Photic hypersensitivity in the premonitory phase of migraine – a positron emission tomography study. Eur J Neurol.

[8] Risal A, Manandhar K, Holen A, Steiner TJ, Linde M (2016). Comorbidities of psychiatric and headache disorders in Nepal: implications from a nationwide population-based study. J Headache Pain.

[9] Serafini G, Pompili M, Innamorati M, Gentile G, Borro M, Lamis DA, Lala N, Negro A, Simmaco M, Girardi P, Martelletti P (2012). Gene variants with suicidal risk in a sample of subjects with chronic migraine and affective temperamental dysregulation. Eur Rev Med Pharmacol Sci.

[10] Signer SF, Lapierre YD (1989). Photophobia in depression. Am J Psychiatry.

[11] Llop S, Frandsen JE, Digre KB, Katz BJ, Crum AV, Zang C, Warnger JEA (2016). Increased prevalence of depression and anxiety in patients with migraine and interictal photophobia. J Headache Pain.

[12] Anagnostou E, Vikelis M, Tzavellas E, Ghika A, Kouzi I, Evdokimidis I, Kararizou E (2016). Photophobia in primary headaches, in essential blepharospasm and in major depression. Int J Neurosci.

[13] Headache Classification Committee of the International Headache Society (2013). The International Classification of Headache Disorders, 3rd edition (beta version). Cephalalgia.

[14] Lovibond PF, Lovibond SH (1995). The structure of negative emotional states: Comparison of the Depression Anxiety Stress Scales (DASS) with the Beck Depression and Anxiety Inventories. Behav Res Ther.

[15] Grassini S, Nordin S (2015) Comorbidity in migraine with functional somatic syndromes, psychiatric disorders and inflammatory diseases: a matter of central sensitization? Behav Med 1–9. [Epub] 10.1080/08964289.2015.1086721 26431372

[16] Seggie J, Canny C, Mai F, McCrank E, Waring E (1989). Antidepressant medication reverses increased sensitivity to light in depression: preliminary report. Prog Neuropsychopharmacol Biol Psychiatry.

[17] Minen MT, De Dahem OB, Van Diest AK, Power S, Schwedt TJ, Lipton R, Silbersweig D (2016). Migraine and its psychiatric comorbidities. J Neurol Neurosurg Psychiatry.

[18] Bakes A, Bradshaw CM, Szabadi E (1990). Attenuation of the pupillary light reflex in anxious patients. Br J Clin Pharmacol.

[19] Kojima M, Shioiri T, Hosoki T, Kitamura H, Bando T, Someya T (2004). Pupillary light reflex in panic disorder. A trial using audiovisual stimulation. Eur Arch Psychiatry Clin Neurosci.

[20] Cascardi M, Armstrong D, Chung L, Paré D (2015). Pupil response to threat in trauma-exposed individuals with or without PTSD. J Trauma Stress.

[21] Cortez M, Rea N, Hunter L, Digre K, Brennan K (2016) Altered pupillary light responses in migrainous photophobia. OR1. doi:10.1111/head.12832 10.1177/0333102416673205 PMC5495574 28387133

[22] Shepherd AJ, Joly-Mascheroni RM (2016) Visual motion processing in migraine: enhanced motion after-effects are related to display contrast, visual symptoms, visual triggers and attack frequency. Cephalalgia. [Epub ahead of print] 10.1177/0333102416640519 27106927

[23] Chapman CR, Bradshaw DH, Donaldson GW, Jacobson RC, Nakamura Y (2014). Central noradrenergic mechanisms and the acute stress response during painful stimulation. J Psychopharmacol.

