# Erratum to: Psychiatric comorbidities and photophobia in patients with migraine

**DOI:** 10.1186/s10194-017-0739-9

**Published:** 2017-03-13

**Authors:** Stefan Seidel, Roland Beisteiner, Maike Manecke, Tuna Stefan Aslan, Christian Wöber

**Affiliations:** Department of Neurology, Medical University of Vienna, Währinger Gürtel 18-20, A-1090 Vienna, Austria

## Erratum

The original publication [1] contains an error in the 2nd paragraph of the introduction and one error in legend of Table 1.

The sentence should be: “These specific characteristics may significantly contribute to the psychosocial impairment and altered quality of life of these subjects.”

The correct table legend can be found below: “DASS Depression Anxiety Stress Scale, N/A not applicable, NRS numeric rating scale, SD standard deviation. + Mann–Whitney-U-test; * chi square test”.
